# A review on application of ultrasound and ultrasound assisted technology for seed oil extraction

**DOI:** 10.1007/s13197-022-05359-7

**Published:** 2022-01-31

**Authors:** R. C. N. Thilakarathna, Lee Fong Siow, Teck-Kim Tang, Yee Ying Lee

**Affiliations:** 1 School of Science, Monash University Malaysia, Jalan Lagoon Selatan, Bandar Sunway, 47500 Subang Jaya, Selangor Malaysia; 2 Institute of Bioscience, University Putra Malaysia, 43400 Serdang, Selangor Malaysia; 3 Monash Industry Palm Oil and Education Research Platform, Monash University Malaysia, Jalan Lagoon Selatan, Bandar Sunway, 47500 Subang Jaya, Selangor Malaysia

**Keywords:** Combination, Fatty acid profile, Oil extraction, Physiochemical properties, Ultrasound

## Abstract

Oil has extensively been extracted from oil-bearing crops and traded globally as a major food commodity. There is always a huge demand from the fats and oils industries to increase oil yield because of profitability benefits. If extraction is conducted under mild operating conditions to preserve and improve the oil quality, then it would be an added value. Ultrasound that works on the cavitational action helps to fulfil the gap. Ultrasound is gaining tremendous interest as an alternative to replace the current conventional extractions approach because of its multiple benefits. Cavitation generated by ultrasound eases the release of oil from cell matrices, thereby allowing the extraction to be carried out under mild processing conditions. The effect enhances the oil yield whilst preserving the quality of the oil. In ultrasound, green solvents can be used to replace toxic organic solvents. Recent up-to-date approaches utilised a combination of ultrasound with enzyme, microwave and supercritical technology to further enhance the oil extraction. This review highlights a comprehensive work of the impact of ultrasound and ultrasound in combination with other technologies on oil extraction, which emphasises the extraction yield and physicochemical properties of the oil, such as fatty acid composition, oxidative stability with the retention of the lipophilic phytochemicals and iodine, saponification values and colour parameters. Understanding of ultrasonication techniques for oil extraction served to be essential and useful information for the fats and oils scientists from academia and industries to explore the possibility of employing a sustainable and mild approaches for extracting oil from various crops.

## Introduction

Presently, several crops, including seeds of vegetables, fruits, flowers and oil seeds, are extensively grown to extract its oil that can be used for edible purposes as food ingredients. According to the United States Department of Agriculture (2021), global oilseed production in 2020/2021 stands at 600 million tons. Seed oils are used widely in different food applications such as cooking or salad oils and confectionery fats. In cases where the extracted oil is rich in essential fatty acids like omega 3, 6 and any phytochemicals, it will be a competitive advantage and profitable to the industries as they can be used as nutraceuticals and sold at a higher price (Putnik et al. 2018).

Until recently, extraction of oil from oilseeds was performed using conventional methods via solvent, steam distillation or mechanical pressing. Of these extraction techniques, solvent extraction techniques are highly dependent on the diffusion of solvent to the plant cell wall (Rodrigues and Fernande 2017). In fact, it is technically and scientifically challenging to handle. Solvent extractions require massive consumption of non-environmental friendly and toxic organic solvents that lead to waste generation. It is also complex and requires long operating hours to operate, which is energy-intensive. Furthermore, extra purification steps are needed to remove the solvent and purify the final product to ensure it is safe for consumption. More intently, the prolonged operating conditions may degrade some of the lipophilic bioactive compounds or unsaturated fatty acid in oil, negatively affecting the quality of the extracted oil. Thus, the shortcomings of existing classical separation methods have forced the industries to search for green extraction techniques that can reduce energy consumption and wastes as well as to replace organic solvents with alternative green solvents whilst improving the yield and quality of the oil (Putnik et al. 2018).

**Fig. 1:**
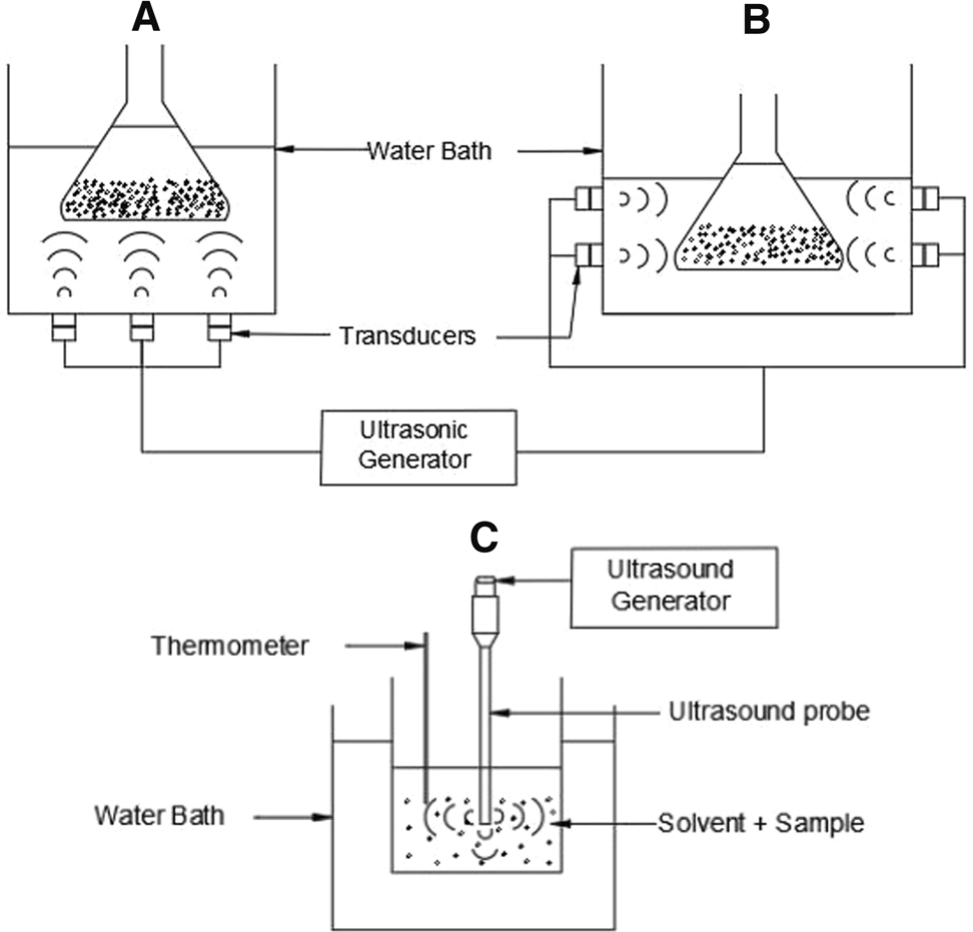
Schematic diagrams of ultrasound extraction methods, ultrasonic bath where transducers are fixed **A** vertically **B** horizontally and, **C** ultrasonic probe. The ultrasonic probe is directly immersed in the sample and solvent mixture while samples are immersed in a steel tank equipped with one or more transducers in the ultrasound bath, resulting in lesser intensity. The intensity given by the probe tip is relatively high due to the small surface area, and probe diameter, length, and shape of the tip directly influence the extraction. Generally, the probe system is preferable over the bath in the extraction industry due to its high efficiency. Either way, ultrasound can be combined with enzymatic, microwave, and supercritical extractions to improve its productivity

One such green technique that is becoming popular in the industries today is ultrasound assisted extraction (UAE). Ultrasound application works on the principle of cavitation or oscillation phenomenon via either using ultrasound probe or ultrasound bath that operates at 20 kHz and 40 kHz frequencies, respectively (Fig. 1). Ultrasonic waves generate vibrations that can create voids that transfer energy to solid particles immersed in the extraction. In addition, cavitation bubbles grow closer to the solid surface and collapse at a higher amplitude forcing the cell wall to rupture, further accelerating the transfer of desired compounds trapped inside into the solvent medium (Senrayan and Venkatachalam 2019). Chemat et al (2017) described in detail the effect generates through independent or combined mechanisms in between fragmentation, erosion, sono-capillarity, sonoporation, shear forces, and detexturation. Cavitation created by ultrasound outperformed conventional extraction approaches (Senrayan and Venkatachalam 2020).

In fats and oils application, ultrasound technology has been employed to extract oil from oilseeds and other promising crops. It was shown to be able to increase oil extraction yield as well as promote the extraction of phytochemicals (Jalili et al. 2018; Rojo-Gutiérrez et al. 2021; Senrayan and Venkatachalam 2019, 2020; Stevanato and Silva 2019). UAE offers several benefits as compared to the conventional methods. It requires a simple and easy instrumental setup. UAE is also environmentally friendly and economically feasible to be adopted. It requires less amount of solvent for the extraction, low energy throughput, and is highly efficient in extracting oil (Rodrigues and Fernandes 2017). More importantly, the mild operational conditions enable retention of minor lipophilic compounds such as phytochemicals, thus, adding value to the oil besides improving its stability. With its mild operating conditions, UAE can also help to prevent the deterioration of the oil. In a more advanced situation, ultrasound can be combined with other technologies like microwave, enzymatic and supercritical fluid extractions to be used directly in the process or could be used as a pre-treatment process. These hybrid techniques are useful in increasing the extraction efficiency of oil (Hu et al. 2020; Goula et al. 2018; Liu et al. 2020).

Looking at the several advantages offered by the ultrasound technology, it is important to understand the use of ultrasound for oil extraction purposes that outperforms other conventional extraction methods. Thus, this review aims to provide a comprehensive insight into the potential of adopting ultrasound application along with ultrasound assisted technologies and oil quality compared to other traditional extraction techniques in major oil seeds and some other promising crops. Therefore, this review is useful to harness the knowledge of UAE applied for oilseed extraction to address the current limitations of traditional extraction approaches used in the fats and oils industries.

## The effect of ultrasound assisted extraction on the oil yield

Multiple studies have been conducted on using UAE for seed oil extraction. Table 1 shows the optimum UAE conditions for the extraction of different types of seed oil such as canola, commonly used vegetables, fruits and flower seed oils with oil yield ranging from 8 to 83% (Table 1).

**Table 1:** The operating and optimized conditions of ultrasound assisted oil extraction of different types of seeds

Matrix	Operating conditions					Method optimization (Yes/No) Optimized Conditions	Formula of oil yield (Y%) or efficiency (E%)	References
	Treatment (frequency and power)		Solvent/sample ratio (v/w) mL/g	Temperature, time combination	Solvent ratio			
Canola seeds	35 kHz, 800 W		5, 10, 15	35,45, 55 °C 30,60, 90 min	Hexane: hexane isopropanol 3:2	Yes- Box-Behnken design 30.66% efficiency at 55 °C, 69.5 min and 9.12% v/w	E% = Oil W/Sample W	Jalili et al (2018)
Vegetable seeds								
Pumpkin seeds	20 kHz, 400 W		10	25–100% amplitude 5–30 min	Hexane 100%	Yes- Response Surface method (RSM) 62.46% yield, at 26.34 min and 89.02% amplitude	Y% = Oil W/Oil W-bearing material	Hernández-santos et al (2016)
Radish seeds	25 kHz, 165 W		4, 8, 12	30, 45, 60 °C 20, 40, 60 min	Ethanol 100%	Yes- Box-Behnken design 25% efficiency at 60 °C, 60 min and 12:1 mL/g	E% = Oil W/Sample W	Stevanato and Silva (2019)
Moringa seeds	20 kHz, 348 W		5–20	30, 40, 50, 60 °C 5, 15, 30, 60, 90 min	Hexane: 100%	Yes- Central Composite Design 53.101% yield at 26. 3 min and 17.8:1 mL/g	Y% = Oil W/Sample W	Mohammadpour et al (2019)
	40 kHz, 200 W		8	30 °C 5, 10, 15 min	Pet ether 100%	No 35.8% yield at 30 °C, 5 min	Y% = Oil W/Sample W	Zhong et al (2018)
Chilli pepper seeds	20 kHz, 200 W		10	50 °C, 50 min	Hexane 100%	No 83.52% efficiency at 50 °C for 50 min	E% = Oil W/ (Sample dry W × crude fat)	Ma et al (2019)
Kapok seeds	24 kHz, 400 W		5–13	40–45 °C 5–15 min	Hexane 100%	Yes- F-CCD statistical design 28.51% yield at 44 °C, 10 min and 13:1 mL/g	Y% = Oil W/Sample W	Senrayan and Venkatachalam (2020)
Fruit seeds								
Papaya seeds	24 kHz, 400 W		5, 10,15	40, 45, 50 °C 5, 10, 15 min	Hexane 100%	Yes- F-CCD statistical design 27.87% efficiency at 48,7 min,1.13 g/mL and 80% amplitude	E% = Oil W/Sample W	Senrayan and Venkatachalam (2019)
	40 kHz, 235–700 W		6–10	5–30 min=	Hexane 100%	Yes- Central composite design (SCCD design) 23.3% yield at 62.5 °C,700 W, 38.5 min,7:1 mL/g	Y% = Oil W/Sample W	Samaram et al (2015)
Grape seeds	20 kHz, 50,100 and 150 W		8	30 min	Hexane 100%	No 14.08% oil yield at 150 W	Y% = Oil W/Sample W	Porto et al (2013)
Raspberry seeds	40 kHz and 250 W		40	30, 40, 50, 60 °C 10, 20, 30 min	Ethanol 100%	Yes-RSM 22.79% yield at 54 °C, 34 min	Y% = Oil W/dry W of sample	Teng et al (2016)
Pomegranate seeds	50–60 Hz, 800 W		5, 10, 20	45, 60, 75 °C 20, 40, 60 min	Hexane	Yes- Box-Behnken design 17.64% yield at 45 °C,10 min, 5:1 mL/g	Y% = Oil W/Sample W	Rojo-Gutiérrez et al (2021)
Sweet Passion (*Passiflora alata* Curtis) seeds	37 kHz, 165 W		2, 5, 10, 15, 20	30, 60 °C 30, 60 min	Ethanol	No 20.96% yield at 60 °C for 60 min, 2:1 mL/g	Y% = Oil W/Sample W	Pereira et al. (2017)
Flower seeds								
Sunflower seeds	24 and 40 kHz		4, 8, 12, 16, 20	20, 30, 40, 50, 60 °C	n-hexane, acetone, ethanol, methanol	Yes, single factorial design 45.4% yield at 50 °C, 2 h, 12:1 mL/g	Y% = Oil W/Sample W	Moradi et al (2018)
Safflower seeds	300 W		2, 3, 4, 5, 6, 7	10, 20, 30, 40 and 50 °C 5, 10, and 15 min	Ethyl acetate 100% n-hexane 100% Ethanol 100% Pet ether 100%	Kinetic model 27.02% efficiency at 35 °C, 30 min, 5 with n-hexane	E% = Oil W/Sample W	A. J. Hu et al (2012)
*Calophyllum inophyllum* *Linn* seeds	40 kHz,190 W, 210 W and 230 W		15, 20, 25	35, 40, 45 °C 15, 20, 25 min	Hexane 100%	Yes- Box-Behnken design 56.2% efficiency at 42 °C, 21 min and 21:1 mL/g	E% = Oil W/Sample W	Fuad et al (2016)

E% = Efficiency %; Y% = Yield%, W = weight

Mechanism of the ultrasound for oil extraction could be influenced by parameters such as extraction time, temperature, sample to solvent ratio, ultrasound power, and type of the solvent. A table to summarise the influence of these parameters is tabulated in Table 2. Apparently, each parameter is cumulatively affecting the ultrasound where it acts positively to increase oil yield and seldom negatively. In addition, literature profoundly emphasizes a considerable extent for each parameter where prolonged action is undesirable on cavitation.

**Table 2:** Impact of processing parameters on UAE

Parameter	Key Findings	Impact on Ultrasound	References
Extraction time	Increasing the extraction time correlates with elevating the oil yield The favourable UAE time is in the range of 5–30 min where the rate of oil extraction accelerates with time	Ultrasonic waves significantly impact the first stage of oil extraction because the intensity of solvent penetration and subsequent dissolution of oil compounds is massive at the initial phase of ultrasonic cavitation, reducing the extraction time However, the accelerating coefficient decreases with time, as the mass transfer rate between the oil and the medium decreases once the equilibrium is reached	Stevanato (2019) Zhang et al. (2016)
	Most influencing parameter in extracting oil from *Moringa oleifera* L. seeds and *Moringa peregrina* seed oil		Thirugnanasambandham (2018) Mohammadpour et al. (2019)
Temperature	Increasing temperature, correlates with increasing the oil yield The favourable UAE temperature range is 30–65 °C	Often, high temperatures tend to lower the viscosity and surface tension of the solvent, thus increasing its permeability and solubility into the cell matrix Ultrasound waves at increased velocity (due to increased temperature) accelerates the release of intracellular oil compounds into the solvent medium	Stevanato (2019) Faud et al. (2016)
	Temperature and time both are the most influencing factors to extract raspberry seed oil		Teng et al. (2016)
	Contradictorily, high temperatures reduce the oil yield	The vapour pressure accelerates at elevated temperatures, the surface tension tends to reduce. Consequently, more cavitation bubbles will develop, but the bubbles implode with a lower intensity. Thus, a less gradient of pressure is developed between inside and outside of bubbles, thus lowering the mass transfer of fat globules	Mohammadpour et al. (2019)
Ultrasound power/Amplitude	Increasing the amplitude of ultrasound result in accelerated extraction efficiency of oil Favourable frequency range is from 20–40 kHz and amplitude often around 400 W	Ultrasound voids that travel through the liquid medium makes the bubbles collapse more violently when subjected to a high amplitude of ultrasonic pressure. As a result, the implosion of cells likely accelerates the fragmentation of fat globules. In fact, this increased power could maximize the collapsing radius 10 times more	Hernández-santos et al. (2016) Hosseini et al. (2015)
	However, frequency of the ultrasound behaves differently where the low frequency is preferred over high frequency	Because the low oscillation causes cavitation bubbles with a short life and collapse violently (transient cavitation), where high power generates stable gas bubbles to stay longer (stable cavitation)	Mushtaq et al. (2020)
Solvent to sample ratio	Increasing solvent/sample ratio increases oil yield Approximately 10–20 ml/g of solvent/sample range gives the best results	A high gradient of concentration between sample and solvent increases the mass transfer, which drives the force of extraction However, prolonged diffusion towards the interior tissues would not increase the oil yield due to lower nucleation sites for the cavitation with less solid concentration	Perrier et al. (2017) Stevanato (2019)
	Most significant parameter for the extraction of pomegranate and favela (*Cnidoscolus quercifolius*) seed oils		Rojo-Gutiérrez et al. (2021) Santos et al. (2021)
Type of the solvent	The oil yield of sunflower seed oil varies from hexane > acetone > methanol > ethanol when extracted from ultrasound	The effect of solvent is mainly attributed to the physical properties such as surface tension, vapour pressure and viscosity that affect the cavitation’s propagation and the intensity Due to the polarity to non-polarity of solvents, affinity for the oil and other dissolved compounds changes. Thus, it is better to use a solvent mixture to get best results	Moradi et al. (2018)
	Around 65% and 67% of rapeseed oil yields using isopropanol and hexane as solvent, respectively when UAE is used. However, only 23%, 50% and 58% of oil were extracted from ethanol, isopropanol and hexane without pretreatment of ultrasound		Perrier et al. (2017)
	Moringa seed oil from methanol is low when compared to chloroform and petroleum ether for UAE		Thirugnanasambandham (2018)

A recent study by Senrayan and Venkatachalam (2020) compared UAE with traditional solvent extraction (SE) in an orbital shaker and Soxhlet extraction (SXE) for the extraction Kapok seed oil. The study revealed a 92.29% of oil recovery within 10 min using UAE, which is significantly shorter than SE and SXE that required 5.7 h and 8 h, respectively. The energy consumption by UAE is 80 fold lower than SXE and 50 fold less than SE. A yield of 70.93% of castor oil within a 9 min of extraction time using a solvent containing mixture of isopropanol: methanol (1:3 w/w) was reported by Naveenkumar and Baskar (2019). Similarly, around 27.02% of safflower seed oil was extracted using UAE whilst, classical method resulted in 25% yield. Despite the almost similar oil yield obtained, UAE was conducted under mild condition of 35 °C and liquid–solid ratio of 5/1 (w/w) within 30 min whereas the traditional approach was performed under elevated conditions of 50 °C and liquid–solid ratio of 6/1 (w/w) for 60 min (Hu et al. 2012). Also, *Moringa Oleifera* seed oil extracted with UAE showed comparable yield as SE which is around 35.78 and 35.26%, respectively. However, extraction time of UAE was 20 min while conventional approach spent around 50 min (Zhong et al. 2018). Contradictorily, oil extraction of cactus pear seeds favoured the soxhelt extraction with a yield of 9.3–9.5% whilst UAE acquired only 5.4–5.6% (Loizzo et al. 2019). However, a majority of studies clearly verify that UAE is able to increase the oil yield using a considerably shorter extraction time, milder conditions and lower energy consumption than conventional oil extraction method.

### The effect of ultrasound assisted enzymatic extraction (UAEE) on the oil yield

Enzymatic extraction is combined with ultrasound extraction in UAEE. Enzymatic extraction is manifested by mechanical crushing, and use enzymes to degrade complexes composed of lipoproteins, lipopolysaccharides, and cell wall to release oil. Non-oil components separated from complex partition into the extractant and oil forms another phase (Song et al. 2019). Since enzymes need more time to break cell wall and this time lapse can be overcome by ultrasound. Thus, it can be seen that enzymes in the medium act to soften and promote the degradation of seed tissues which enhances the solvent permeability. Ultrasonic waves create bubble cavitation, and altogether, enzymes and ultrasound work to improve the oil extractability.

The operating and optimized conditions of UAEE of different types of seeds are illustrated in the Table 3. Typically, the ultrasound probe is immersed in the solvent bath containing enzymes (cellulase, pectinase and hemicellulase) and oilseeds. Also, mixture of enzymes is more effective than using them individually. In this regard, UAEE was employed for extraction of pomegranate seed oil (Goula et al. 2018), date seed oil (Amigh and Dinani 2020), and pine kernel oil (Chen et al. 2016). Significantly, Goula et al. (2018) emphasised the positive impact of the ultrasonication in aiding the enzymatic oil recovery. The study revealed that ultrasound increased the enzymatic extraction of pomegranate seed oil by 18.4% and decreased the extraction time by 91.7%. Furthermore, with the aid of ultrasound, the enzyme–substrate interaction was improved mainly because of the demolishment of the tissues created by ultrasound that increased in the mass transfer of solvent-enzyme into the cellular structure and the elevated contact surface area between the tissue’s enzyme-aqueous solution and the seed powder. In addition, Amigh and Dinani (2020) highlight that besides the effect of ultrasound, having a cooking process of seed powder before extraction could enhance the extraction yield.

**Table 3:** The operating and optimized conditions of Ultrasound assisted enzymatic extraction of different types of seeds

Matrix	Ultrasound operating conditions			Enzyme treatment				Results		References
	Direct probe /bath /pretreatment	Frequency/power	Time & Temperature	Enzyme type	Enzyme concentration	Medium and solvent/solid ratio	Incubation	Oil yield at optimized conditions	Oil recovery	
**Pomegranate seeds**	Direct probe	20 kHz, 130 W	3–60 min 55 °C	Cellulase Peclyve V	2–4% w/w	Aqueous 2/1–6/1 mL/g	Incubated while ultrasonication	18.15 g oil/100 g at 55 °C, 10 min, 2% w/w of peclyve V and 6/1 mL/g	95.8%	Goula et al (2018)
**Date seeds**	Direct probe	1000 W	1 h 55 °C	Cellulase	1.5% w/w	Aqueous 2/1–10/1 mL/g	Incubated while ultrasonication	9.5% at 2/1 mL/g for 40 min	-	Amigh and Dinani (2020)
**Pine (*Pinus pumila*) seeds**	Direct probe	20 kHz, 900 W	1.2, 1.5, 1.8 h 40, 50, 60 °C	Cellulose, hemicellulase, pectinase, acid protease, neutral protease, alkaline protease and α-amylase	0.5–3.5% w/w	Aqueous 4, 6, 8 mL/g	Incubated while ultrasonication	30.96% at 1:1:1 w/w/w of cellulose, pectinase: hemicellulose	-	F. Chen et al. (2016)
***Sapindus mukorossi* seeds**	In bath	40 kHz, 150–250 W	30–60 min 50–60 °C	Protease, cellulase, pectinase, hemicellulase, α-glucosidase	2–4% w/w	Aqueous 8–16 mL/g	Incubated while ultrasonication	37.97% at 240 W, 60 °C,56 min, 16/1 mL/ g, 4% of (1:1:1 w/w/w) Protease, cellulase, and pectinase	82.67%	Z. Liu et al (2019)
**Perilla seeds**	As a pretreatment	100–350 W	5–50 min 20–70 °C in hexane	Cellulase, Viscozyme L, Alcalase 2.4L, Protex 6L, and Protex 7L	Manufacturer’s dosage	Aqueous 6/1 mL/g	At 50 °C for 120 min	Highest yield in cellulase (50 °C, 30 min, 250 W)	81.74%	Li et al (2014)
**Peanut**	As a pretreatment	250 Hz	0–70 min 45 °C in hexane	Cellulase	1.47% w/w	Aqueous buffer	At 56 °C for 120 min	27.24% at 1.2% w/w for 42.4 min	-	Haji Heidari and Taghian Dinani (2018)
**Walnut**	As a pretreatment	250 Hz	0–70 min 45 °C in hexane	Cellulase	0–2% w/w	Aqueous buffer	At 56 °C for 60–240 min	29.23% at 2% w/w,47.37 ultrasound min and 110.9 incubation min	-	Zahra et al (2018)

Oil yield = $\frac{{{\text{Mass}}\;{\text{of}}\;{\text{extracted}}\;{\text{oil}}}}{{{\text{Mass}}\;{\text{of}}\;{\text{initial}}\;{\text{seed}}\;{\text{powder}}}} \times 100\%$; Oil recovery = $\frac{{{\text{Mass}}\;{\text{of}}\;{\text{extracted}}\;{\text{oil}}\;{\text{from}}\;{\text{UAEE}}}}{{{\text{Mass}}\;{\text{of}}\;{\text{oil}}\;{\text{from}}\;{\text{oxhlet}}}} \times 100\%$

Apart from that, UAEE can also be performed by incubating seed powder with an enzyme and subsequently submerging the mixture in the ultrasonic bath. Although the ultrasound is applied indirectly, the physical effect of ultrasonic waves effectively enhances enzyme–substrate reaction. Following that, oil extracted from *Sapindus mukorossi* seed kernel oil could reach up to 82.67% in the enzyme mixture of neutral protease, cellulase and pectinase (Liu et al. 2019). However, the long incubation time needed for enzymes to react with seed powder which may take around 8–12 h, is the major drawback of this method.

Alternatively, ultrasound was used as a pretreatment prior to applying the enzymatic hydrolysis method for the extraction of peanut, perilla seeds, and walnut oil (Haji Heidari and Taghian Dinani 2018; Li et al. 2014; Zahra et al. 2018). Pretreatment works to disrupt the cell wall of the solid matrix and to reach the oil bodies before enzymatic extraction. Usually, ultrasound pretreatment time ranges from 30 to 50 min. Subsequent enzymatic extraction can extend up until 120 min. Nevertheless, the duration of extraction time is still far lesser than the extraction time used in convectional enzymatic extraction.

In general, UAEE can be considered as a cost-effective and environmentally friendly approach for oil extraction. However, sufficient data is yet available to fairly compare all the types of UAEE.

### The effect of ultrasound assisted supercritical extraction (UASE) on the oil yield

Supercritical fluid extraction (SFE) is a sophisticated method using supercritical fluid for oil extraction. The supercritical fluid is a specific liquid or gas formed as a homogeneous fluid when the temperature and pressure increased above the critical point. The demarcation surface between liquid and gas is disappeared under supercritical conditions and the infusibility, and the density of the fluid lies between gas and liquid. Therefore, a slight increase in pressure drastically improves the supercritical fluid's infusibility and solvating power, which result in high extractability (Salinas et al. 2020).

However, the extraction efficiency of SFE is always hindered by the low solubility of the oil in CO_2_ fluid because of deviation of the polarity. As a remedy, SFE can be assisted with ultrasound. Cavitation generated from ultrasound resulted in micro jetting of supercritical fluid into the cell wall matrix which enhances the penetration of CO_2_ fluid. The assistance of the ultrasound reduces the SFE operating parameters such as temperature, pressure, CO_2_ flow rate, and time. The operating and optimized conditions of UASE of different types of seeds are represented in Table 4. UASE performed on passion fruit seed oil produced an oil yield of 20.6% compared to SFE that only yields 12.3% at 40 °C of temperature and 16 MPa of pressure (Barrales et al. 2015). The authors suggest that a lower combination of pressure and temperature enables formation of CO_2_ gas bubbles inside the extracting bed under the ultrasound vibrations resulting in cavitation. Also, using ultrasound as a pretreatment for hemp seeds (Da. Porto et al. 2015) and *Iberis amara* seeds (Liu et al. 2020) have shown a similar effect in oil increment. The studies indicate that nearly 10 min of ultrasound pretreatment is sufficient rather than prolong treatment in order to get maximum oil yields. Noticeably, Dias et al (2019) followed ultrasonication of umbu seed powder after treating with SFE first and achieved the highest oil yield compared to SFE or ultrasound extraction alone. The authors assume that the breaking of non-polar materials like waxes and resins with subsequent matrix modification will lead to enhance the extraction. In as much as the ability of ultrasound to increase the yield while reducing the severity of operating parameters is significant, integrating ultrasound with other extractions is useful.

**Table 4:** The operating and optimized conditions of ultrasound assisted supercritical extraction of different types of seeds

Matrix	Ultrasound operating conditions			Supercritical fluid extraction conditions				Oil yield at best conditions	References
	Mode of application	Frequency/power	Time & Temperature	CO_2_ fluid (F)/seed (S) ratio	Flow rate	Time & temperature	Pressure		
Passion fruit seeds	Direct	20 kHz 160, 400, 640 W	20 min 38–53 °C	210 kg/1 kg (F/S)	1.75 × 10^–4^ kg/s	100 min	16–29 MPa	20.6% at 26 MPa, 40 °C and 160 W	Barrales et al (2015)
Almond seeds	Direct	19–20 kHz 110 W	0–3.5 h 40–60 °C	0.468 g/mL (S/F)	10–15 kg/h	Same with ultrasound	200–320 bar	~ 20% at 45 °C 280 bar, and 12.5 kg/h	Riera et al (2010)
*Iberei Amara* flower seeds	Pretreatment	25 kHz 0.625,1.25,2.5 W/mL In hexane	5–10 min	–	3 mL/min	55 °C and 80 min	25 MPa	25.28% at 1.25 W/mL from 10 min	X. Liu et al (2020)
Hemp seeds	Pretreatment	20 kHz 200 W	10, 20, 40 min 25 °C	45 kg/kg	8 × 10^−5^ kg/s	40 °C and 4 h	300 bar	24.03% from 10 min	Da. Porto et al (2015)
Umbu seeds	Post treatment	20 kHz 500 W Ethanol/water mixture	4 min	–	11.66 g/min	40 °C and 180 min	15,20,30 MPa	10.9%	Dias et al (2019)

Oil yield = $$\frac{Mass\,of\,extracted\,oil}{Mass\,of\,initial\,seed\,powder}\times 100\%$$

### The effect of ultrasound assisted microwave extraction (UAME) on the oil yield

Microwave irradiation is an advanced technology used to enhance the extraction process. Microwaves are generated at a frequency range of 300 MHz to 300 GHz, and these electromagnetic radiations penetrate the oil bodies converting energy into heat. The resultant intra-cellular pressure causes the swelling of the cell and ruptures the cell wall as well as the lipoprotein membrane surrounded by the individual lipid body. Thus, the release of oil is depending upon the heating rate (Fouad et al. 2018).

As for UAME, the effect generated by microwaves is localised in plant cells and its driving force is combined with the acoustic cavitation produced by ultrasonic voids. The resulted larger surface area due to small particle size caused by the implosion of the cell wall helps to enhance the mass transfer rate and intraparticle diffusion, boosting the oil extractability. UAME showed a promising result in reducing energy consumption. A summarized data of operating and optimized conditions employed to extract different seed oils by ultrasound assisted microwave extraction is shown in Table 5.

**Table 5:** The operating and optimized conditions of Ultrasound assisted microwave extraction of different types of seeds

Matrix	Mode of application	Operating conditions				Oil yield at best conditions	References
		Ultrasound power	Microwave power	Time & Temperature	Solvent/solid ratio		
Tea seeds	Microwave and ultrasound transducers together	25 kHz 300–600 W	2450 MHz 200–500 W	20–40 min 70–90 °C	8 mL/g	31.52% at 70 °C, 38 min, 440 W in ultrasound and 550 W in microwave	B. Hu et al (2019a, b)
Coffee beans	Ultrasonic-microwave cooperative extractor	100–500 W	100–500 W	30–70 °C	10–30 mL/g	10.58% 10 min, 28 mL/g	Q. Chen et al (2020)
*Allanblackia parvifora* seeds	Sequential treatment (First ultrasonication, secondly microwave)	20 kHz 150 W	187.58 W	60 min, 40 °C 120 s	9 mL/g	62.95%	Quaisie et al (2021)
Canola seeds	Microwave pretreatment	12.05–33.9 W/cm^−2^	607 W for 5 min	30, 60, 90 min 311 K	17, 22, 28, 34 mL/g	45% at 60 min,57% amplitude	Sánchez et al (2019)

Oil yield = $$\frac{Mass\,of\,extracted\,oil}{Mass\,of\,initial\,seed\,powder}\times 100\%$$

UAME utilised 38 min whilst SE needed 6 h to extract a similar amount of tea seed (*Camelia sinensis*) oil (Hu et al. 2019a, b). Chen et al. (2020) developed a UMAE technique to extract 10.58% of green coffee bean oil. The study further demonstrated that elevation of temperature from 40 to 70 °C induces the emanation oil from coffee beans. Nevertheless, evidence on the break-down of constituents in the oil by microwave and ultrasound have been reported (Hosseini et al. 2015). The hybrid method studied by Quaisie et al. (2021) confirmed a higher oil yield of 64.15% and extraction efficiency of 92.16% for *Allanblackia parvifora* seed oil with a 33.33% reduction in extraction time over the conventional method. The synergetic effect of both technologies again ensured by Sánchez et al. (2019) as the yield of canola oil increased when seeds are pretreated with microwaves followed by ultrasound extraction. In another study, around 85.23% of tiger nut (*Cyperus esculentus* L.) oil was extracted using UAME in combination with aqueous enzymatic extraction using cellulase, pectinase and hemicellulose at dosage of 1/1/1, (w/w/w) (Hu et al. 2020).

## The effect of ultrasound technology on fatty acid composition (FAC), oxidative stability and physiochemical properties of extracted oil

Apart from improving extraction yield, the quality of oil extracted remains a critical and utmost important parameter for the fats and oils industries regardless of any of the extraction processes being employed. Therefore, the extraction techniques that can preserve or improve oil quality will be of much preference. In this regard, the influence of ultrasound treatment on the quality of extracted oil was reviewed in this section which is mainly based on the evaluation on changes of the FAC and oxidative stability, which is indicated by the peroxide value (PV), p-anisidine (AV), acid value, Totox value (TV) and induction time. Oil with low acidity, PV (primary oxidation), AV (secondary oxidation) confer better oxidative and hydrolytic stability. In addition, other physiochemical properties such as iodine value, saponification value and colour parameters are also compared with convectional extraction.

In terms of FAC, the majority of the studies demonstrated that the UAE has slight effect on the fatty acid profile of the oil extracted (de Mello et al. 2017; Hernández-santos et al. 2016; Senrayan and Venkatachalam 2019; Sicaire et al. 2016). Interestingly, sunflower and kiwi seed oils extracted by UAE showed a significant increment in linolenic acid (ω-3) acid (Moradi et al. 2018; Cravotto et al. 2011).

On the other hand, pumpkin seed oil extracted from the UAE had a high oxidative stability with a low PV, AV and TV (Hernández-santos et al. 2016). Samaram et al (2014, 2015) and Zhang et al. (2019) confirmed the same finding on the high oxidative stability of papaya seed oil extracted from UAE. The promising results may be due to the mild conditions (lower temperature and shorter extraction time) used in UAE than conventional SE. In addition, it prevents the degradation of oil, particularly those with unsaturated fatty acid and deterioration of antioxidants, thereby protecting the oil from undergoing auto-oxidation. Also, the partial destruction of cell membrane promotes the release of more oil inherent bioactive compounds. Another rationale behind improving the oil stability may be due to the mechanical and thermal inactivation of oxidative enzymes such as peroxidase and lipase generated by ultrasound voids. A recent study by Stevanato and Silva (2019) demonstrated the longest oxidative induction time of 72.5 min for radish seed oil extracted by UAE, while classical extraction presented with the oxidative stability of only 27.5 min. Traditional means may have caused thermal degradation of oil due to an extended period of exposing the oil under elevated temperature. In contrast, Sicaire et al. (2016) stated that the UAE extracted rapeseed oil showed a slight increase in peroxide value (0.53 meq O_2_ / kg oil) and conjugated dienes. The authors further suggested performing the UAE under a modified inert atmosphere in the absence of oxygen or in the presence of argon to prevent oxidation of oil.

Ultrasound combined with other technologies also shows similar results. A study of pine kernel oil extracted via UAEE also reported similar FAC compared to SE except for a slight increment in acid value, but is still under the edible range (F. Chen et al. 2016). Additionally, slight differences in FAC were reported for kenaf seed oil when differentiating the type of solvent in the ethanol, hexane, and aqueous mediums for UAEE (Zhang et al. 2020). Similarly, the FAC of Cumbaru oil extracted using supercritical fluid in the presence or absence of ultrasound showed to have no significant difference (Dos Santos et al. 2016). Liu et al. (2020) found that *Iberis amara* seed oil extracted using ultrasound as a pretreatment for supercritical extraction had a better FAC profile with a slight increase in MUFA and a small decrease in SFA. The PV of oil extracted with enzymes in the presence and absence of ultrasound pre-treatment reported for peanuts did not vary significantly (Haji Heidari and Taghian Dinani 2018). Contradictorily, Zahra et al (2018) imply that ultrasound pretreatment increases the PV of walnut seed oil.

A comparison of iodine value, saponification value, acid value and colour parameters of different seed oils extracted based on ultrasound and ultrasound assisted other technologies are represented in Table 6. Regardless the ultrasound assisted extraction mechanism, iodine, saponification, and colour values are not influenced by the ultrasound treatment frequently. However, acid and peroxide values have been significantly affected as discussed above. Often, ultrasonication operated at mild conditions (summarized in Table 2) have resulted in lowering acidity and generation of peroxides compared to conventional methods conducted at elevated levels. From the authors point of view the influence of ultrasound on FAC, PV, AV, TV including other physiochemical parameters is more prominent when combined with other extraction technologies. This is merely because, when ultrasound voids are used along with another approach, the resulting force is more effective than used alone. However, more comparative research data is essential to confirm the effect.

**Table 6:** Comparison of physiochemical parameters of different types of seed oils between ultrasound and convectional extraction methods

Matrix	Iodine value (Wiji’s) (gI/100 g)		Saponification value (mg KOH/g)		Acid value (mg KOH/g)		Peroxide value		Colour		References
	UAE	SXE	UAE	SXE	UAE	SXE	UAE	SXE	UAE	SXE	
Kapok seeds	102.23	101.46	184.73	186.34	1.56	1.66	–	–	32Y,5.5R	46Y,4.5R	Senrayan and Venkatachalam (2020)
Papaya seeds	71.00	71.18	–	–	–	–	–	–	–	–	Samaram et al (2015)
	61.73	64.3	209.07	153.96	1.26	1.61	1.03(meq /kg)	1.35 (meq /kg)	3, 9.5,—(L*, a^*^, b^*^)	8, 50,—(L*, a^*^, b^*^)	Zhang et al (2019)
Sunflower seeds	–	–	193.3	192.4	0.85(% oleic acid)	0.83	15.53(meq /kg)	14.66 (meq /kg)	-	-	Moradi et al (2018)
Chillie seed oil	129.83	130.15	188.4	191.21	3.7	4.0	1.52 mmol/kg	1.77 mmol/kg	60.07, -0.59, 1.01 (L*, a^*^, b^*^)	60.67, -0.57, 0.97 (L*, a^*^, b^*^)	Ma et al (2019)
Moringa seeds	78.67	71.69	–	–	–	–	3 mmol/kg	4 mmol/kg	–	–	Mohammadpour et al (2019)

	UAEE	SXE/CN	UAEE	SXE/CN	UAEE	SXE/CN	UAEE	SXE/CN	UAEE	SXE/CN	
*Sapindus mukorossi* seeds	113.15	107.39	191.83	189.54	4.12	4.45	–	–	–	–	Z. Liu et al (2019)
Pine (*Pinus pumila*) seeds	153.5	148.89	196.46	196.12	2.88	2.72	5.25 mmol/kg	6.16 mmol/kg	–	–	F. Chen et al. (2016)
Perilla seeds	192	190	192	193	4.3	4.6	3.7 mmol/kg	3.5 mmol/kg	–	–	Li et al (2014)
Peanut	–	–	–	–	–	–	3.5(meq /kg)	3.43(meq /kg)	4.44, -3.59, 10.63 (L*, a^*^, b^*^)	3.16, -3.75, 9.18 (L*, a^*^, b^*^)	Haji Heidari and Taghian Dinani (2018)
Walnut	151.07	145.28	–	–	–	–	1.02(meq /kg)	1.04(meq /kg)	28.22, -0.95, 22.9 (L*, a^*^, b^*^)	28.22, -1.58, 23.4 (L*, a^*^, b^*^)	Zahra et al (2018)

	UASE	SXE	UASE	SXE	UASE	SXE	UASE	SXE	UASE	SXE	
*Iberei Amara* flower seeds	113.87	105.57	198.98	198.25	3.74	4.12	2.97(meq /kg)	3.87(meq /kg)	–	–	X. Liu et al (2020)

	UAME	SXE	UAME	SXE	UAME	SXE	UAME	SXE	UAME	SXE	
Tea seeds	87.43	88.29	195.73	194.38	3.54	4.28	13.18(meq /kg)	16.57(meq /kg)	–	–	B. Hu et al (2019a, b)
*Allanblackia parvifora* seeds	32.74	32.68(ME)	173.44	168.77(ME)	0.34	0.40(ME)	2.17(meq /kg)	3.5(ME)	59.81, -4.98, 16.89 (L*, a^*^, b^*^)	60.52, -6.18, 21.43 (L*, a^*^, b^*^)	Quaisie et al (2021)

UAE, Ultrasound assisted extraction; UAEE, Ultrasound assisted enzymatic extraction; UASE, Ultrasound assisted supercritical extraction; UAME, Ultrasound assisted microwave extraction; SXE, Soxhlet extraction; ME, Mechanical extraction; CN, Control

## The effect of ultrasound on the polyphenolic content of extracted oil

Ultrasound technology promotes the extraction of lipophilic antioxidants and pigments. Porto et al (2013) found that grape seed oil extracted using UAE under a nitrogen atmosphere had higher polyphenols, total tannins, total anthocyanins, cinnamic acids, flavanols and antioxidant activity. Again, ultrasound extracted pumpkin peel/seed oil has more antioxidants (tocopherols, phytosterols, and β-carotene) than SE (Bovo et al. 2019). The high polyphenolic compounds extracted was attributed by the acoustic cavitation where the mechanical effect of ultrasound increased the release of active compounds. In contrast, the antioxidant activity of *Moringa oleifera* seed oil extracted by ultrasound and conventional extraction methodologies did not show any appreciable differences (Zhong et al. 2018). Also, when grape seed powder is treated with ultrasound twice, the total polyphenol content of the oil decreases (Porto et al. 2013). It was suggested that the double ultrasound treatment for UAE is not desirable for recovery of phenolic compounds as prolonged application may reason for the degradation of active compounds. Similarly, virgin olive oil extracted under sonication demonstrated an increment of tocopherols by 60%, carotenoids and chlorophylls by 30% whilst the total polyphenols decreased by 30% for Coratina type of olive (Clodoveo et al. 2013). The authors assumed that the polyphenols decrement can be ascribed by activity of enzymes like peroxidase that is still preserved during the sonication time in Coratina type of olive. This is because ultrasound waves can influence both enzyme activation and inactivation during the extraction process. Suggesting the same behaviour, due to the effect of ultrasound, grape seed oil shows a higher phenolic compound content and antioxidant capacity by the free radical method, but a lower ability to scavenge DPPH radical than the control (Böger et al. 2018). Conversely, the milder processing parameters of UAE, together with a controlled atmosphere are favourable to enhance the extraction of bioactive compounds in the extracted oil.

Moreover, high contents of total tocopherol (830.51 mg/kg), carotenoids (57.06 mg/kg), phenols (104.22 mg GAE/kg), sterols (1.77 g/kg) and Trolox equivalent antioxidant capacity (1.38 µmol Trolox/g) were obtained when ultrasound is employed for supercritical fluid extraction of *Iberis amara* seed oil (Liu et al. 2020). Further, microwave extraction assisted with ultrasound promotes the extraction of carotenoids and chlorophyll to produce a phenolic rich olive oil (Tamborrino et al. 2019). Interestingly, H. Li et al (2017) revealed that the best antioxidant capacity for perilla seed oil using UAEE compared to SE, occupying 615.25 and 410.5 mg GAE/kg of total phenols, respectively.

## Industrial potential and future trends

The application of ultrasonication on an industrial scale is currently restricted due to the need of large capital to install high power/amplitude units. Lack of technical know-how immensely impacts small and medium scale oil industries. Even though many studies have been conducted on the laboratory scale, the findings disclose via pilot plants to scale up are limited. Despite these challenges, ultrasound outperforms the existing conventional methods in the industry. For instance, the organic solvents used in soxhlet extractors can be replaced with green solvents, where in cases when the green solvents are used in conventional approach that it may resulted in low oil yield. However, to work ultrasound properly, seeds have to be ground into a smaller size, where at an industrial scale, it would be disadvantageous due to additional processing steps. Nevertheless, refining the extracted oil is a significant process conducted in the oil industry to meet the desired quality parameters. However, there is considerable time and energy consumption behind this complex treatment. Advances of ultrasound suggest that impurities of the extracted oil can be minimized based on the reduction of pigments and colour parameters in the extracted oil (Samaram et al. 2014; Hosseini et al. 2015). More research is essential to link refining of oil with UAE where there is no reported data. As the oil industry is always looking forward to advanced extraction techniques with high efficiency, low cost, fewer impurities, and sustainable development, UAE for edible oil is a better solution. As the newest trend, ultrasound coupled with both microwave and enzymatic aqueous extraction of cherry and tiger nut seed oil has given oil recovery greater than 80% with superior oil quality than soxhelt extraction (Hu et al. 2019a, b; Hu et al. 2020). The results ensure the effect of ultrasonication hybrid with other technologies is more significant, even to replace solvents with an aqueous medium for oil extraction. Recently, the sequential effect of ultrasonication and microwave extractions studied by Quaisie et al (2021) suggest that there is a significant effect when applying ultrasonication first, followed by microwave extraction. Thus, a new research area is open to considering the sequence of combining ultrasonication with other seed oil extraction methods. In the future, most industries will embrace sustainability, and conventional methods will fail to compete with the market where advanced technologies like ultrasound are used.

## Conclusion

UAE is an efficient and recent extraction technique that can be used either directly or combined with other novel methods such as enzymatic, supercritical, and microwave to replace the conventional oil extraction methods. UAE can replace organic solvents with green solvents or reduce solvent usage and operate under mild operational conditions. Ultrasound cavitation generated by the transducer directly influences the extractability of oil. The intensity and propagation of the cavitation highly depend on extraction time, temperature, ultrasound power, and solvent/sample ratio. Despite few exceptions, the overall increment of oil yield at reduced energy consumption is solid enough to conclude the efficacy of ultrasound application for oil extraction. Remarkably, the quality of the extracted oil remained unchanged in terms of the fatty acid profile and thermal-oxidative stability. Also, UAE can be a promising approach to improve the extraction of lipophilic phytochemical composition.

## Abbreviations

AV: *P*-anisidine value
FAC: Fatty acid composition
PV: Peroxide value
TV: Totox value
SE: Solvent extraction
SXE: Soxhlet extraction
SFE: Supercritical fluid extraction
UAE: Ultrasound assisted extraction
UAEE: Ultrasound assisted enzymatic extraction
UAME: Ultrasound assisted microwave extraction
UASE: Ultrasound assisted supercritical extraction
